# Efficacy and Safety of Pegcetacoplan in Kidney Transplant Recipients With Recurrent Complement 3 Glomerulopathy or Primary Immune Complex Membranoproliferative Glomerulonephritis

**DOI:** 10.1016/j.ekir.2024.09.030

**Published:** 2024-10-10

**Authors:** Andrew S. Bomback, Erica Daina, Giuseppe Remuzzi, John Kanellis, David Kavanagh, Matthew C. Pickering, Gere Sunder-Plassmann, Patrick D. Walker, Zhongshen Wang, Zurish Ahmad, Fadi Fakhouri

**Affiliations:** 1 Division of Nephrology, Columbia University Irving Medical Center, New York, New York, USA; 2 Istituto di Ricerche Farmacologiche Mario Negri IRCCS, Bergamo, Italy; 3 Department of Nephrology, Monash Health and Centre for Inflammatory Diseases, Clayton, Australia; 4 Department of Medicine, Monash Medical Centre, Clayton, Australia; 5 National Renal Complement Therapeutics Centre, Newcastle University, UK; 6 Imperial College, London, UK; 7 Division of Nephrology and Dialysis, Department of Medicine III, Medical University of Vienna, Vienna, Austria; 8 Department of Renal Pathology, Arkana Laboratories, Little Rock, Arkansas, USA; 9 Apellis Pharmaceuticals, Inc., Waltham, Massachusetts, USA; 10 Lausanne University Hospital, Centre Hospitalier Universitaire Vaudois, Lausanne, Switzerland

**Keywords:** C3 glomerulopathy, complement, immune complex membranoproliferative glomerulonephritis, pegcetacoplan, proteinuria

## Abstract

**Introduction:**

Complement 3 glomerulopathy (C3G) and primary immune complex membranoproliferative glomerulonephritis (IC-MPGN) have high risks for disease recurrence and allograft loss in transplant kidneys. Pegcetacoplan (targeted complement 3 [C3]/C3b inhibitor) may prevent excessive deposition of C3 and complement 5 [C5] breakdown products and associated renal damage.

**Methods:**

NOBLE (NCT04572854) is a prospective, phase 2, multicenter, open-label, randomized controlled trial evaluating the efficacy and safety of pegcetacoplan in posttransplant patients with recurrent C3G or IC-MPGN. The primary end point was reduction in C3c staining on renal biopsy at week 12 for patients who received either pegcetacoplan 1080 mg twice weekly by subcutaneous infusion plus standard-of-care (SOC) or SOC only.

**Results:**

Ten patients received pegcetacoplan and 3 received SOC only through week 12. At week 12, 5 of 10 pegcetacoplan-treated patients (50%) achieved ≥2 orders of magnitude (OOM) reduction in C3 staining (4 of these 5 had 0 staining and absent electron microscopy deposits) and 8 of 10 (80%) achieved ≥1 OOM reduction; 1 of 3 (33%) SOC-only patients showed staining reduction. Mean C3G histology activity score decreased by >54% in 8 of 10 pegcetacoplan-treated patients (80.0%). Pegcetacoplan-treated patients with baseline urine protein-to-creatinine ratio (uPCR) ≥1000 mg/g showed a median (interquartile range [IQR]) 54.4% (–56.33 to –53.95) reduction in proteinuria at week 12. In addition, pegcetacoplan-treated patients showed stable estimated glomerular filtration rate (eGFR), reduced plasma sC5b-9, and increased serum C3. Pegcetacoplan was well-tolerated and most adverse events were mild/moderate. No discontinuations, treatment withdrawals, or deaths were reported.

**Conclusion:**

NOBLE demonstrated efficacy, safety, and tolerability of pegcetacoplan for patients with posttransplant recurrent C3G and primary IC-MPGN.

## Graphical abstract

**Figure:**
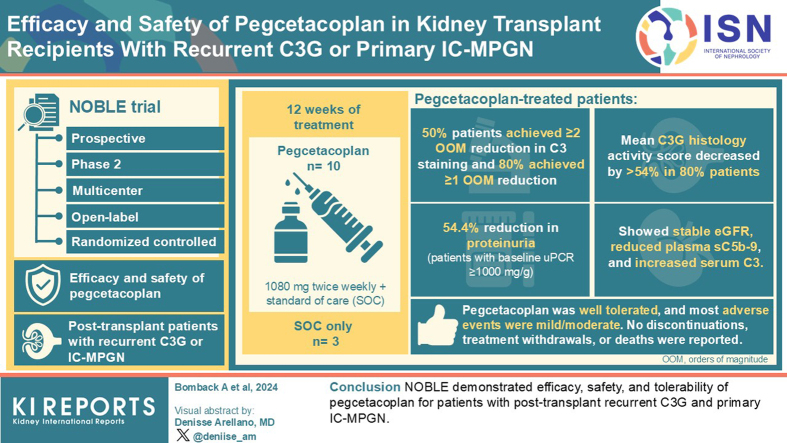

See Commentary on Page 7

C3G and primary IC-MPGN are rare glomerulopathies, with an estimated incidence of <5 cases per million people,^1^^,^^2^ and have a poor prognosis, with a median time to kidney failure of approximately 10 years from diagnosis.^3^^,^^4^ Renal transplantation is an option for patients who reach end-stage kidney disease, but the incidence of disease recurrence after transplant is high (up to 80% of patients),^5^^,^^6^ with up to 50% of patients losing their renal allografts due to disease recurrence within 5 to 10 years.^7^, ^8^, ^9^, ^10^, ^11^, ^12^, ^13^ Currently, there are no approved treatments for C3G or IC-MPGN.^7^^,^^10^

C3G and primary IC-MPGN are caused by dysregulated complement activation which results in glomerular deposition of C3 and C5 breakdown products^14^ and subsequent kidney damage.^7^ Therefore, C3 inhibition is a rational therapeutic approach for these glomerulopathies. Pegcetacoplan is a targeted C3 and C3b inhibitor that prevents activity of both C3 and C5 convertases through the classical, lectin, and alternative complement activation pathways.^15^ Pegcetacoplan has been approved by the US Food and Drug Administration and the European Medicines Agency since 2021 for the treatment of paroxysmal nocturnal hemoglobinuria.

The objective of the phase 2 NOBLE trial was to evaluate the efficacy and safety of pegcetacoplan for improving the underlying pathophysiology of C3G or primary IC-MPGN in patients with posttransplant disease recurrence. Here, we describe the primary 12-week findings of the NOBLE trial.

## Methods

### Study Design

**Figure 1:**
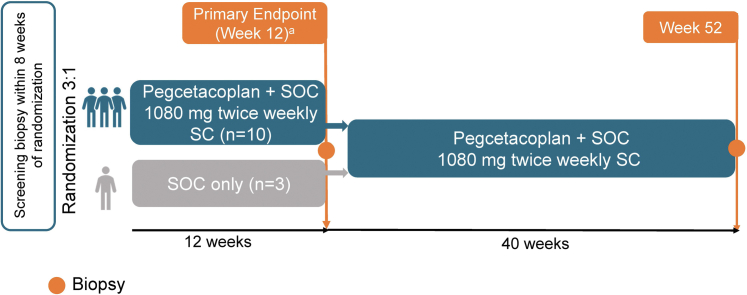
Study design. OOM, orders of magnitude; SC, subcutaneous; SOC, standard-of-care. Note: Kidney biopsy was performed during the screening period, within 8 weeks of randomization. ^a^Proportion of patients with reduction in renal biopsy C3c staining (defined as a decrease of ≥2 OOM) at week 12 from baseline.

NOBLE (NCT04572854) is a prospective, phase 2, multicenter, open-label, randomized controlled trial evaluating the efficacy and safety of pegcetacoplan versus SOC for posttransplant patients with recurrent C3G or primary IC-MPGN. Enrolled patients were randomized 3:1 to receive either pegcetacoplan 1080 mg twice weekly by subcutaneous infusion plus SOC (pegcetacoplan group) or SOC only (SOC-only group) for 12 weeks, followed by a 40-week noncontrolled period during which all patients received pegcetacoplan (Figure 1).

### Patient Selection

Adult patients (aged ≥18 years at screening) with clinical and pathologic evidence of posttransplant recurrent C3G or primary IC-MPGN that was not secondary to another condition (e.g., infection, malignancy, monoclonal gammopathy, autoimmunity, chronic antibody-mediated rejection, chronic thrombotic microangiopathy, or a medication) with at least 2+ staining for C3c on kidney biopsy and an eGFR ≥15 ml/min per 1.73 m^2^ (according to the Chronic Kidney Disease Epidemiology Collaboration [CKD-EPI] Creatinine [2021] equation) were included in NOBLE. The kidney disease must have been stable or worsening in the 2 months preceding the first dose of pegcetacoplan. Patients were excluded from NOBLE if disease in the renal allograft was secondary to another condition that would, in the opinion of the investigator, confound interpretation of the study results. Complete inclusion and exclusion criteria are listed in the Supplementary Methods.

### Outcomes

The primary efficacy end point was the proportion of patients with reduction in C3c immunofluorescence staining (defined as a decrease of ≥2 OOM from baseline) on renal biopsy after 12 weeks of treatment with pegcetacoplan. Key secondary efficacy end points were the proportion of patients with stabilization or improvement in eGFR (any increase or any decrease ≤25% relative to baseline) over time; the proportion of patients with stabilization or improvement of serum creatinine concentration over time; changes from baseline biopsy in C3c staining over time; and changes and percentage changes from baseline in eGFR and serum creatinine concentration over time. Exploratory efficacy end points included changes in proteinuria over time and changes in key biopsy features over time (including activity score [range: 0–21] and chronicity score [range: 0–10] based on C3G histologic index). The complete list of exploratory end points is provided in Supplementary Methods. Safety end points included the number, incidence, severity, and seriousness of treatment-emergent adverse events (TEAEs), as well as the number and incidence of graft loss and rejection episodes.

### Statistical Analyses

Given the study’s exploratory nature, no formal statistical hypothesis testing was performed. Therefore, the sample size was not based on the statistical power of the study. Up to 12 patients were planned to be enrolled in this study. Categorical variables are presented as number (percentage) of patients, and descriptive statistics are used to summarize continuous variables, including number of patients, mean, and SD, and median and IQR. A *post hoc* analysis of the primary end point using observed data at baseline and week 12 was performed with the Cochran-Mantel-Haenszel test.

## Results

### Patient Disposition and Baseline Characteristics

**Figure 2:**
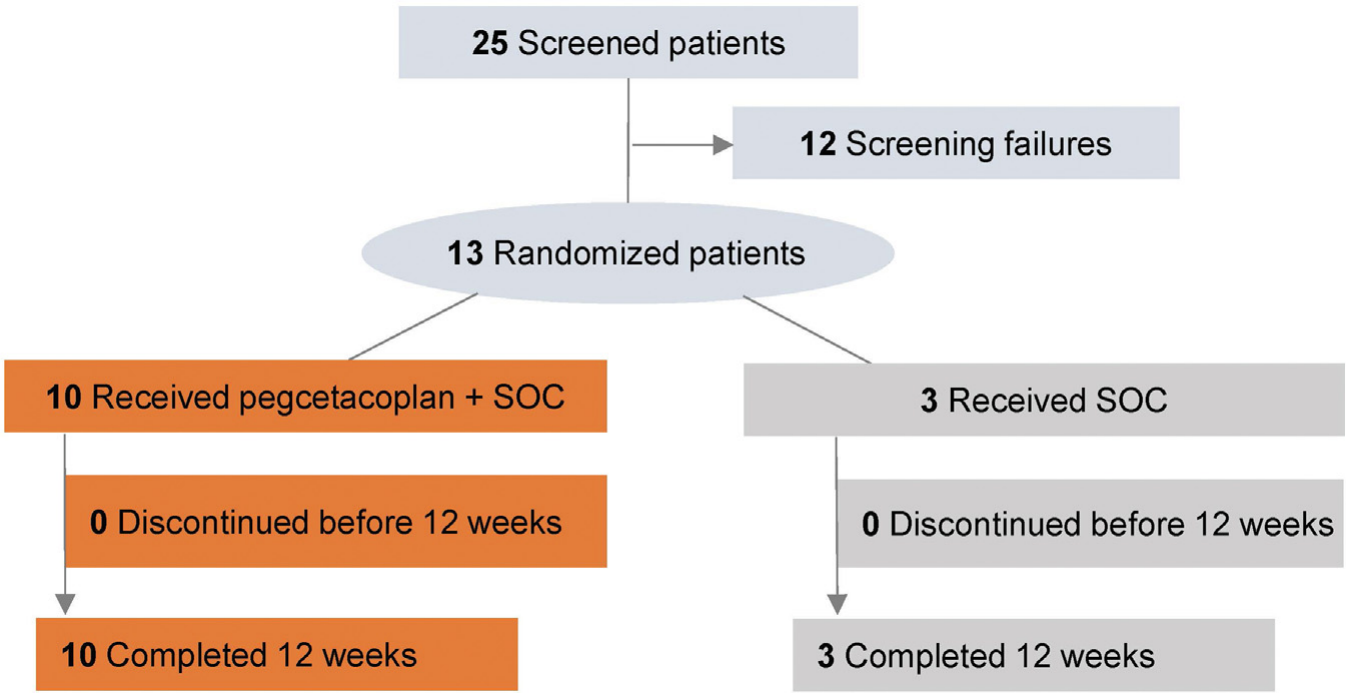
CONSORT flow diagram. SOC, standard-of-care.

A total of 25 patients were screened and 13 patients were enrolled in NOBLE: 10 patients in the pegcetacoplan-plus-SOC group and 3 patients in the SOC-only group (Figure 2; Table 1). Of the 10 patients in the pegcetacoplan group, 8 had C3G and 2 had primary IC-MPGN based on the screening biopsy. Of the 3 patients in the SOC-only group, 2 had C3G and 1 had primary IC-MPGN based on the screening biopsy.

**Table 1:** Baseline patient demographics and disease characteristics

Parameter	Pegcetacoplan group (*n* = 10)	SOC-only group (*n* = 3)	Overall (*N* = 13)
Age at screening, yrs^a^			
Mean (SD)	39.8 (12.59)	44.3 (24.03)	40.8 (14.80)
Median (min, max)	35.0 (20, 64)	43.0 (21, 69)	36.0 (20, 69)
Sex, *n* (%)^a^			
Female	5 (50.0)	1 (33.3)	6 (46.2)
Male	5 (50.0)	2 (66.7)	7 (53.8)
Race, *n* (%)^a^			
White	9 (90.0)	3 (100)	12 (92.3)
Not reported	1 (10.0)	0	1 (7.7)
Ethnicity, *n* (%)^a^			
Hispanic or Latino	1 (10.0)	1 (33.3)	2 (15.4)
Not Hispanic or Latino	9 (90.0)	2 (66.7)	11 (84.6)
Underlying disease, *n* (%)^a^			
C3G	8 (80.0)	2 (66.7)	10 (76.9)
C3GN	5 (50.0)	2 (66.7)	7 (53.8)
DDD	2 (20.0)	0	2 (15.4)
Undetermined	1 (10.0)	0	1 (7.7)
Primary IC-MPGN	2 (20.0)	1 (33.3)	3 (23.1)
Total number of kidney transplants, *n* (%)^a^			
1	7 (70.0)	2 (66.7)	9 (69.2)
2	2 (20.0)	1 (33.3)	3 (23.1)
3	1 (10.0)	0	1 (7.7)
Time since most recent kidney transplant, yrs^a^			
Mean (SD)	1.9 (1.32)	5.5 (0.52)	2.7 (1.96)
Median (min, max)	1.5 (0.65, 4.89)	5.7 (4.88, 5.82)	2.40 (0.65, 5.82)
Time since most recent posttransplant disease recurrence, yrs^a^			
Mean (SD)	1.0 (0.89)	2.8 (2.42)	1.4 (1.48)
Median (min, max)	0.8 (0.17, 3.10)	2.4 (0.58, 5.37)	0.9 (0.17, 5.37)
Triplicate spot urine uPCR, mg/g^a^			
Mean (SD)	1523.4 (1035.83)	2314.8 (2170.68)	1706.1 (1307.84)
Median (IQR)	1688.8 (506.67– 2185.00)	1711.3 (509.67–4723.33)	1703.7 (509.67– 2185.00)
Triplicate spot urine uPCR ≥1000 mg/g proteinuria, mg/g, n^a^	6	2	8
Mean (SD)	2236.8 (599.48)	3217.3 (2129.81)	2481.9 (1053.92)
Median (IQR)	2047.7 (1703.67–2963.67)	3217.3 (1711.33–4723.33)	2047.7 (1707.50–2973.83)
eGFR, ml/min per 1.73 m^2^^a^^,^^b^			
Mean (SD)	50.9 (12.73)	53.3 (11.37)	51.5 (12.01)
Median (IQR)	47.0 (41.0–65.0)	50.0 (44.0–66.0)	50.0 (42.0–65.0)
Serum creatinine, mg/dl^a^			
Mean (SD)	1.6 (0.32)	1.5 (0.15)	1.5 (0.29)
Median (IQR)	1.7 (1.4–1.7)	1.5 (1.3–1.6)	1.6 (1.4–1.7)
Serum albumin, g/dl^a^			
Mean (SD)	4.1 (0.54)	3.6 (0.45)	4.0 (0.53)
Median (IQR)	4.0 (3.70–4.50)	3.6 (3.20–4.10)	3.9 (3.60–4.50)
Serum C3, mg/dl^a^			
Mean (SD)	56.1 (33.83)	103.0 (67.88)	64.6 (41.67)
Median (IQR)	70.0 (42.0–76.0)	103.0 (55.0–151.0)	70.0 (42.0–84.0)
Plasma soluble C5b9, ng/ml^c^			
Mean (SD)	651.4 (1024.68)	145.3 (41.28)	534.6 (914.89)
Median (IQR)	241.0 (226.0–645.0)	122.0 (121.0–193.0)	232.0 (165.0–318.0)
C3c staining, orders of magnitude, *n* (%)^a^			
0	0	0	0
1	0	0	0
2	1 (10.0)	1 (33.3)	2 (15.4)
3	9 (90.0)	2 (66.7)	11 (84.6)
Total activity score, mean (SD)^a^	8.4 (6.72)	3.3 (5.77)	7.2 (6.66)
Total chronicity score, mean (SD)^a^	1.0 (2.31)	4.7 (1.15)	1.8 (2.61)
Vaccination prior to randomization, *n* (%)^d^			
*Haemophilus influenzae*	10 (100.0)	3 (100.0)	13 (100.0)
*Streptococcus pneumoniae* (PCV13)	8 (80.0)	2 (66.7)	10 (76.9)
*Streptococcus pneumoniae* (PPSV23)	7 (70.0)	3 (100.0)	10 (76.9)
*Neisseria meningitidis* (A, C, W, and Y)	9 (90.0)	3 (100.0)	12 (92.3)
*Neisseria meningitidis* (B)	9 (90.0)	3 (100.0)	12 (92.3)
Previous therapies, *n* (%)^d^^,^^e^			
Renin-angiotensin system blocking agents	8 (80.0)	3 (100.0)	11 (84.6)
Systemic glucocorticoids	9 (90.0)	2 (66.7)	11 (84.6)
MMF/MPS	10 (100.0)	3 (100.0)	13 (100.0)
Calcineurin inhibitors^f^	10 (100.0)	3 (100.0)	13 (100.0)
Concomitant therapies, *n* (%)^d^^,^^g^			
Systemic antibacterial agents	5 (50.0)	0	5 (38.5)
Systemic glucocorticoids	4 (40.0)	0	4 (30.8)
Renin-angiotensin system blocking agents	3 (30.0)	0	3 (23.1)
Calcineurin inhibitors^f^	3 (30.0)	0	3 (23.1)
MMF	2 (20.0)	0	2 (15.4)
G-CSF	1 (10.0)	0	1 (7.7)

C3, complement 3; C3G, complement 3 glomerulopathy; C3GN, complement 3 glomerulonephritis; C5b9, complement 5b9; DDD, dense deposit disease; eGFR, estimated glomerular filtration rate; G-CSF, granulocyte-colony stimulating factor; IC-MPGN, immune complex membranoproliferative glomerulonephritis; IQR, interquartile range; MMF, mycophenolate mofetil; MPS, mycophenolate sodium; PCV13, pneumococcal 13-valent conjugate vaccine; PPSV23, pneumococcal polysaccharide 23-valent vaccine; SOC, standard-of-care; uPCR, urine protein-to-creatinine ratio.

a Intention-to-treat set.

b Calculated using the Chronic Kidney Disease-Epidemiology Collaboration (CKD-EPI) Creatinine equation (2021). If confirmation of the result was required, the CKD-EPI creatinine-cystatin C equation was used.

c Pharmacodynamic set.

d Safety set.

e Medications used within 12 weeks of screening.

f Includes tacrolimus, ciclosporin.

g Medications used from the time of informed consent through the end-of-study visit.

Before study entry, 9 patients had 1 kidney transplant, 3 had 2 kidney transplants, and 1 had 3 kidney transplants (Table 1). The median (minimum, maximum) time since the most recent kidney transplant to day 1 of the trial was 1.5 (0.65, 4.89) years in the pegcetacoplan group and 5.7 (4.88, 5.82) years in the SOC-only group. The median (minimum, maximum) time of disease recurrence (defined as the time between the first posttransplant biopsy to show disease recurrence and trial entry) was 0.8 (0.17, 3.10) years in the pegcetacoplan group and 2.4 (0.58, 5.37) years in the SOC-only group. Evidence of recurrent C3G or IC-MPGN, with at least 2+ staining for C3c, was confirmed in all patients by a screening renal allograft biopsy, within 8 weeks of randomization. Findings were confirmed by a central pathologist.

All patients in both groups completed the study up to week 12, and no patients discontinued treatment for any reason. Median (IQR) compliance with pegcetacoplan treatment was 100% (96%–100%).

### Efficacy End Points

**Figure 3:**
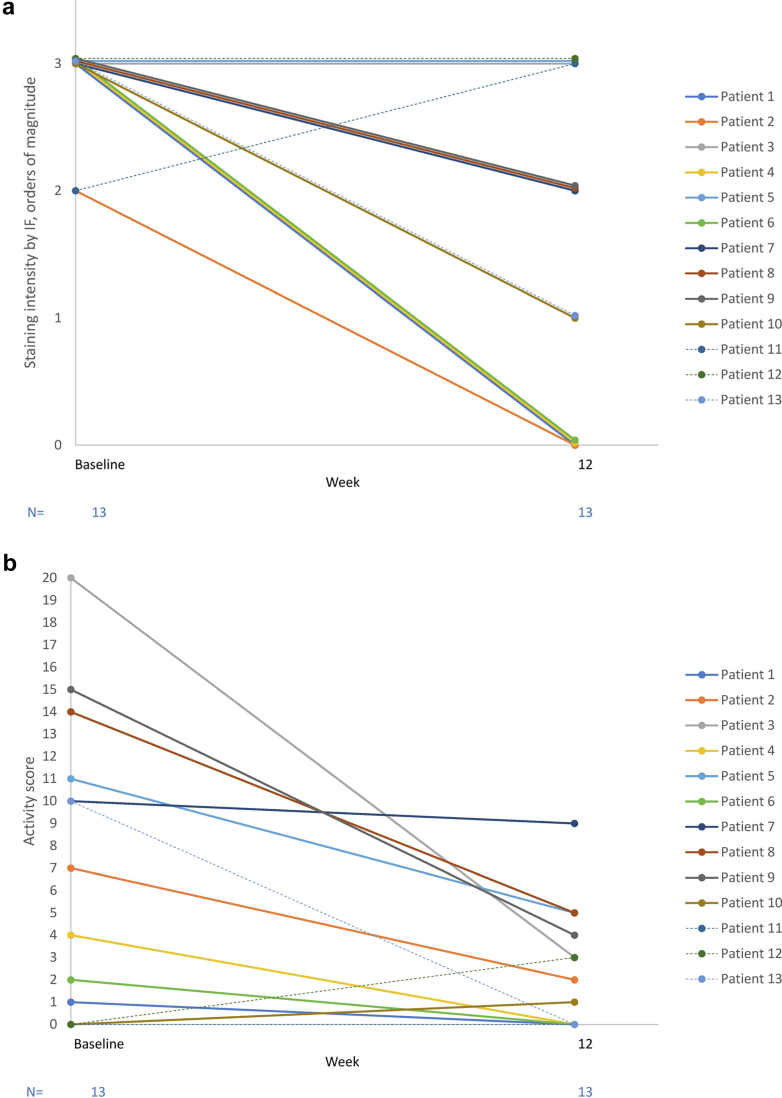
Changes in histologic parameters from baseline to week 12. (a) Changes in C3c staining intensity. (b) Changes in C3G histology activity score. C3G, C3 glomerulopathy; IF, immunofluorescence; SOC, standard-of-care. Note: Solid lines represent patients who received pegcetacoplan plus SOC week 0–12. Dashed lines represent patients who received only SOC weeks 0–12.

At week 12, in the pegcetacoplan group, C3c staining intensity in kidney biopsies decreased significantly from baseline (*P* = 0.0094 as per the Cochran-Mantel-Haenszel test) in a *post hoc* analysis. Five of 10 patients (50%) who received pegcetacoplan showed a reduction in C3c staining of ≥2 OOM, and 8 of 10 (80%) showed a reduction of ≥1 OOM of staining (Supplementary Table S1, Figure 3a). One of 3 patients (33%) in the SOC-only group showed a reduction of ≥2 OOM; the remaining 2 (67%) SOC-only patients showed the same or increased staining at week 12 (Supplementary Table S1, Figure 3a). Four of 5 pegcetacoplan-treated patients with ≥2 OOM reduction in staining achieved 0 C3c staining and had absent electron microscopy (EM) deposits at week 12 (Supplementary Table S1 and Supplementary Figure S1).

At baseline, the median (IQR) activity score of the C3G histologic index was 8.5 (2.0–14.0) for pegcetacoplan-treated patients and 0 (0.0–10.0) for SOC-only patients. At week 12, the activity score decreased to 2.5 (0.0–5.0) for pegcetacoplan-treated patients, indicating reduced disease activity, and remained at 0 (0.0–3.0) for SOC-only patients. Among the 10 pegcetacoplan-treated patients, the activity score decreased by more than 54% in 8 (80.0%) patients, with 3 (30%) achieving a score of 0 at week 12. One pegcetacoplan-treated patient (10%) experienced an increase from 0 to 1 in activity score, and 1 (10%) experienced a decrease from 10 to 9. One SOC-only patient (33.3%) achieved a decreased activity score (the same patient experienced a reduction in C3c staining in the SOC-only group). The remaining 2 (67%) patients in the SOC-only group had an activity score of 0 at baseline (Supplementary Table S1, Figure 3b). Among the 5 pegcetacoplan-treated patients who experienced a decrease in staining intensity of ≥2 OOM, 3 achieved 100% decreases in activity score and 1 achieved a 71% decrease. These same 4 patients also had absent EM deposits at week 12. The remaining patient with a decrease in staining intensity of ≥2 OOM experienced an increase in activity score from 0 to 1.

At baseline, the median (IQR) chronicity score of the C3G histologic index was 0.0 (0.0–0.0) for pegcetacoplan-treated patients and 4.0 (4.0–6.0) for SOC-only patients. At week 12, the chronicity score increased to 1.5 (0.0–6.0) for pegcetacoplan-treated patients, and decreased to 3.0 (3.0–8.0) for SOC-only patients.

**Figure 4:**
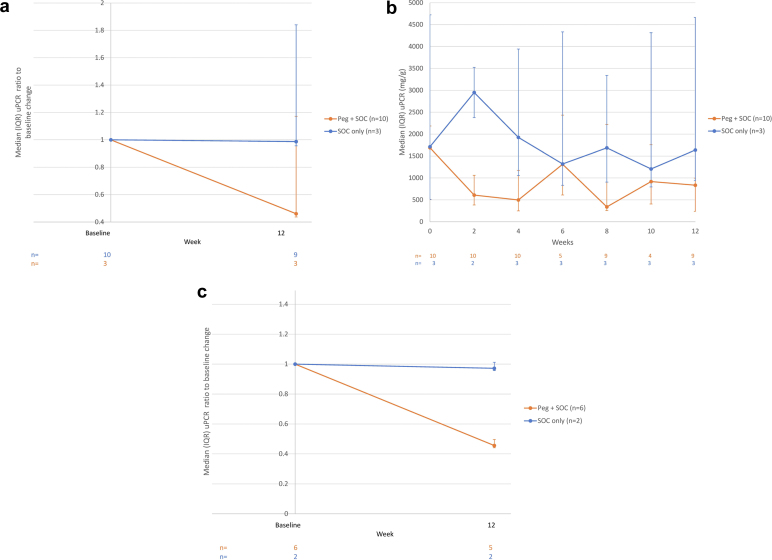
Changes in uPCR from baseline to week 12. (a) Median (IQR) uPCR^a^ (mg/g) ratio to baseline change. (b) Changes in uPCR from baseline to week 12. (c) Median (IQR) uPCR^a^ (mg/g) ratio to baseline change for patients with uPCR ≥1000 mg/g at baseline. ^a^Measured by triplicate first-morning spot urine. IQR, interquartile range; ITT, intent-to-treat; Peg, pegcetacoplan; SOC, standard-of-care; uPCR, urine protein-to-creatinine ratio.

At week 12, treatment with pegcetacoplan resulted in a median (IQR) reduction of 54.0% (–56.33 to 17.19) in uPCR (measured by triplicate first-morning urine) compared with a median (IQR) change of −1.3% (−4.34 to 84.04) with SOC only (Figure 4a and b). Among pegcetacoplan-treated patients, 5 (50.0%) achieved at least a 50% decrease in uPCR. Among SOC-only patients, 1 patient (33%) experienced an 84% increase in uPCR and the remaining 2 (67%) achieved minor decreases (1% and 4%) (Table S1). Among a subgroup of patients with uPCR ≥ 1000 mg/g proteinuria at baseline, those treated with pegcetacoplan (*n* = 5) achieved a median (IQR) 54.4% (−56.33 to −53.95) reduction in uPCR compared with a 2.8% (−4.34 to −1.26) reduction with SOC only (*n* = 2) (Figure 4c).

Kidney function, as measured by eGFR, remained stable over 12 weeks in both groups. In the pegcetacoplan group, median (IQR) eGFR values were 47.0 (41.0–65.0) ml/min per 1.73 m^2^ at baseline and 42.0 (35.0–72.0) ml/min per 1.73 m^2^ at week 12. In the SOC-only group, values were 50.0 (44.0–66.0) ml/min per 1.73 m^2^ at baseline and 41.0 (40.0–61.0) ml/min per 1.73 m^2^ at week 12 (individual values are provided in Supplementary Table S1). Serum creatinine levels were also stable over 12 weeks in both groups. In the pegcetacoplan-treated group, median (IQR) values were 1.7 (1.4–1.7) mg/dl at baseline and 1.6 (1.3–1.8) mg/dl at week 12. In the SOC-only group, values were 1.5 (1.3–1.6) mg/dl at baseline and 1.6 (1.3–1.9) mg/dl at week 12 (Supplementary Table S1).

### Pharmacokinetic, Pharmacodynamic, and Complement Assessments

**Figure 5:**
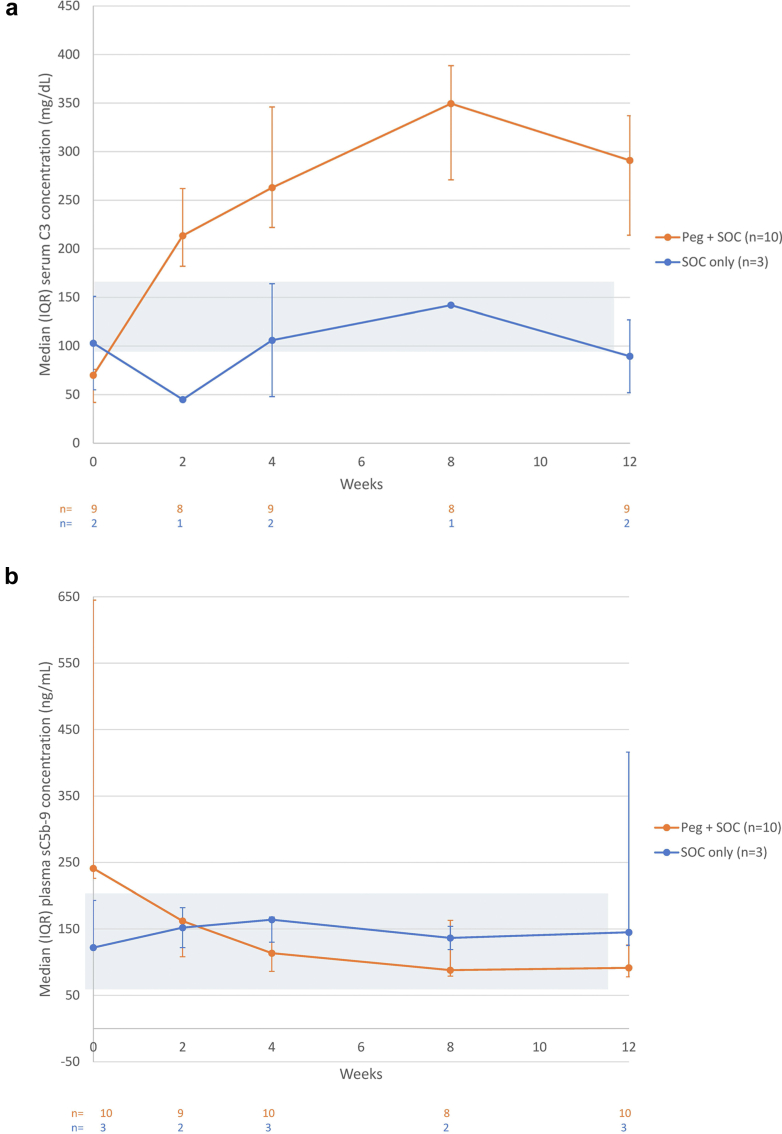
Change in complement biomarkers from baseline to Week 12 for (a) serum C3 and (b) plasma sC5b9. Note: Blue boxes represent normal ranges (serum C3: 94–166 mg/dl; sC5b-9: 59–207 ng/ml). C3, complement 3; IQR, interquartile range; ITT, intent-to-treat; Peg, pegcetacoplan; sC5b9, soluble complement 5b9; SOC, standard-of-care.

Among pegcetacoplan-treated patients, pegcetacoplan concentrations reached steady state between week 4 and week 12, with the maximum mean (SD) trough concentration of 752.44 (121.997) μg/ml observed at week 8 (Supplementary Figure S2). In the pegcetacoplan group, serum C3 increased in all 9 patients who had baseline and week 12 values (Supplementary Figure S3). In the SOC-only group, serum C3 decreased in the 2 patients who had both baseline and week 12 values (Figure 5). At week 12, the median (IQR) serum C3 values were 291.0 (214.0–337.0) mg/dl (normal range, 94–166 mg/dl) for the pegcetacoplan group, an increase of 378.6% from baseline (70.0 [42.0–76.0] mg/dl), and 89.5 (52.0–127.0) mg/dl for the SOC-only group, a decrease of 10.7% from baseline (103.0 [55.0–151.0] mg/dl).

Plasma sC5b-9 decreased in 9 of 10 pegcetacoplan-treated patients (Supplementary Figure S4). The single patient without decreased sC5b-9 had normal concentrations at baseline (87 ng/ml) and week 12 (185 ng/ml). Plasma sC5b-9 increased in all 3 SOC-only patients. Pegcetacoplan treatment reduced median (IQR) baseline plasma sC5b-9 levels to within the normal range (59–207 ng/ml). At week 12, plasma sC5b-9 values were 91.5 (78.0–126.0) ng/ml for the pegcetacoplan group, a decrease of 67.6% from baseline (241.0 [226.0–645.0] ng/ml), and 145.0 (125.0–416.0) ng/ml for the SOC-only group, an increase of 18.9% from baseline (122.0 [121.0–193.0] ng/ml).

### Safety

Overall, 7 patients (70.0%) in the pegcetacoplan group and all 3 patients (100%) in the SOC-only group had at least 1 TEAE (Table 2). Most of the TEAEs were mild or moderate in severity. No TEAEs led to study discontinuation, treatment withdrawal, or death. Three (30.0%) patients in the pegcetacoplan group experienced TEAEs that were possibly related to treatment by the investigator.

**Table 2:** Summary of safety data

Outcome	Pegcetacoplan group (*n* = 10)	SOC-only group (*n* = 3)
Summary of TEAEs, *n* (%)^a^		
Any TEAE	7 (70.0)	3 (100)
Treatment-related TEAEs^b^	3 (30.0)	0
Maximum severity of TEAEs		
Mild	2 (20.0)	1 (33.3)
Moderate	2 (20.0)	2 (66.7)
Severe^c^	3 (30.0)	0
TEAEs leading to dose interruption^d^	1 (10.0)	0
TEAEs leading to treatment discontinuation	0	0
SAEs^e^	5 (50.0)	0
Deaths	0	0
TEAEs by organ class and preferred term, *n* (%)		
Infections and infestations	2 (20.0)	2 (66.7)
COVID-19	1 (10.0)	1 (33.3)
Influenza	1 (10.0)	1 (33.3)
Genital herpes simplex	1 (10.0)	0
Herpes simplex	1 (10.0)	0
Tinea versicolor	0	1 (33.3)
Renal and urinary disorders	4 (40.0)	0
Acute kidney injury	1 (10.0)	0
Loss of bladder sensation	1 (10.0)	0
Nephropathy toxic	1 (10.0)	0
Renal impairment	1 (10.0)	0
Blood and lymphatic system disorders	2 (20.0)	1 (33.3)
Anemia	1 (10.0)	1 (33.3)
Neutropenia	1 (10.0)	0
Gastrointestinal disorders	2 (20.0)	1 (33.3)
Diarrhea	1 (10.0)	1 (33.3)
Nausea	1 (10.0)	0
Investigations	2 (20.0)	0
Cytomegalovirus test positive	1 (10.0)	0
SARS-CoV-2 test positive	1 (10.0)	0
Vascular disorders, *n* (%)	1 (10.0)	1 (33.3)
Hypertension	1 (10.0)	1 (33.3)
General disorders and administration site conditions	1 (10.0)	0
Infusion site rash	1 (10.0)	0
Infusion site swelling	1 (10.0)	0
Immune system disorders	1 (10.0)	0
Transplant rejection^f^	1 (10.0)	0
Injury, poisoning, and procedural complications	1 (10.0)	0
Procedural hypertension	1 (10.0)	0
Procedural pain	1 (10.0)	0
Metabolism and nutrition disorders	1 (10.0)	0
Iron deficiency	1 (10.0)	0
Musculoskeletal and connective tissue disorders	1 (10.0)	0
Spinal stenosis	1 (10.0)	0
Nervous system disorders	1 (10.0)	0
Hypoesthesia	1 (10.0)	0
Sciatica	1 (10.0)	0
Respiratory, thoracic, and mediastinal disorders	1 (10.0)	0
Cough	1 (10.0)	0
SAEs by organ class and preferred term, *n* (%)		
Renal and urinary disorders	2 (20.0)	0
Acute kidney injury	1 (10.0)	0
Nephropathy toxic	1 (10.0)	0
Blood and lymphatic system disorders	1 (10.0)	0
Neutropenia	1 (10.0)	0
Immune system disorders	1 (10.0)	0
Transplant rejection^f^	1 (10.0)	0
Infections and infestations	1 (10.0)	0
Genital herpes simplex	1 (10.0)	0
Musculoskeletal and connective tissue disorders	1 (10.0)	0
Spinal stenosis	1 (10.0)	0

SAE, serious adverse event; SOC, standard-of-care; TEAE, treatment-emergent adverse event.

a A TEAE was defined as an AE that started or worsened after the first pegcetacoplan dose for patients in the pegcetacoplan group or randomization for the SOC group.

b The treatment-related TEAEs were neutropenia, diarrhea, infusion site rash, infusion site swelling, influenza, and acute kidney injury (each occurred in 1 patient).

c One patient had neutropenia, 1 patient had a graft complication that was originally reported as transplant rejection, and 1 patient had sciatica and spinal stenosis; only the neutropenia was considered possibly related to pegcetacoplan by the investigator.

d One patient had neutropenia and SARS-CoV-2 test positive.

e Five patients had 6 unique SAEs. The events of neutropenia (also considered a severe AE) and acute kidney injury were considered possibly related to pegcetacoplan by the investigator but not by the sponsor. The remaining SAEs (graft complication [originally reported as transplant rejection], genital herpes simplex, nephropathy toxic, and spinal stenosis) were considered not related to pegcetacoplan by both investigator and sponsor.

f Graft complication that was originally reported as transplant rejection.

A total of 6 unique serious AEs (SAEs) occurred in 5 of 10 patients (50.0%) in the pegcetacoplan group; no SAEs occurred in the SOC-only group (Table 2). The sponsor considered all 6 SAEs to be not related to pegcetacoplan due to the presence of confounding factors such as underlying diseases and/or use of immunosuppressive treatment that provided an alternative etiology. All SAEs resulted in inpatient hospitalization or prolongation of existing hospitalization. However, no SAEs resulted in death, life-threatening outcome, persistent or significant disability, or permanent damage.

Treatment-related TEAEs were generally consistent with the known safety profile of pegcetacoplan or were related to underlying disease. No encapsulated bacterial infections and no events of rejection and/or graft loss were reported during the first 12 weeks of the study. Two patients in each treatment group experienced infections (Table 2): all were considered mild or moderate, none were related to treatment, and all resolved. No relevant safety trends were observed in laboratory values, vital sign measurements, electrocardiogram data, or physical examinations.

## Discussion

The NOBLE study demonstrated mechanistic and clinical efficacy, safety, and tolerability of pegcetacoplan, a targeted C3 and C3b inhibitor, in patients with posttransplant recurrent C3G and primary IC-MPGN. In these patients, 12-week treatment with pegcetacoplan resulted in statistically significant reduction in C3c staining on renal biopsy; this reduction in staining by immunofluorescence was confirmed by the resolution of deposits by EM. These changes occurred along with a decreased C3G histology activity score, reduced plasma sC5b-9, and increased serum C3. In addition, in patients with uPCR ≥ 1000 mg/g at baseline, pegcetacoplan treatment resulted in a median of 54.4% reduction in proteinuria. Pegcetacoplan was generally well-tolerated, with most TEAEs being mild or moderate severity, and no new safety signals were identified.

Complement dysregulation is the underlying cause of C3G and primary IC-MPGN, and targeted complement inhibitors as treatment for these diseases have achieved varying levels of success. The C5 inhibitor eculizumab has shown limited clinical benefit in patients with native and posttransplant disease, with only some patients achieving sustained response to treatment.^16^, ^17^, ^18^, ^19^, ^20^ The ACCOLADE trial, which evaluated the selective C5a receptor blocker avacopan for patients with native and posttransplant disease did not reach its primary end point of improvement in the histology index of disease activity and showed only partial benefit for reducing C3G progression.^21^^,^^22^

Proximal complement inhibition is also being evaluated for C3G and primary IC-MPGN. Danicopan, an inhibitor of complement factor D, prevents C3 convertase formation in the alternative pathway while leaving the classical and lectin pathways intact.^23^ In 2 phase 2 proof-of-concept studies among patients with native disease, the coprimary end points of change in a composite histology score (activity index, C3c staining, macrophage infiltration, and chronicity index) and a 30% decrease in proteinuria from baseline at 6 and 12 months were not met. Lack of ability to reach adequate levels of alternative pathway inhibition was associated with a lack of efficacy in histologic end points with only a marginal improvement from baseline and no change in C3c staining at 6 and 12 months from baseline and a lack of clinical end point improvement of 30% decrease in proteinuria. Due to this lack of efficacy, the trials were concluded early.^24^^,^^25^

Proximal inhibition with iptacopan, which inhibits Factor B in the alternative pathway and leaves the classical and lectin pathways intact, was evaluated in an open-label phase 2 study of patients with native and posttransplant disease. Although a significant decrease in C3 staining from baseline was observed among posttransplant patients with recurrent disease who received 12 weeks of treatment with iptacopan, thus meeting the trial’s primary end point, no patient achieved 0 C3 staining intensity. Histologic activity index measures and changes to EM deposits were not reported for these patients. Furthermore, there was a nonsignificant reduction in proteinuria (uPCR: 23.9 g/mol at baseline to 17.7 g/mol at day 84; *P* = 0.4766) for transplant recipients.^2^ Twelve-month data for these patients reported eGFR outcomes with sustained serum C3 increases and plasma sC5b-9 decreases but did not discuss C3 staining or proteinuria.^26^

Pegcetacoplan directly inhibits C3 and C3b, the point of convergence for the 3 complement pathways, which contrasts currently available complement inhibitors, as well as those under investigation. By preventing the breakdown of C3 and further deposition of C3 breakdown products in glomeruli, pegcetacoplan directly impacts the complement dysregulation at the root of C3G and primary IC-MPGN.^11^^,^^27^^,^^28^ In the NOBLE trial, efficacy was observed for the 8 pegcetacoplan-treated patients (80%) who achieved decreased staining, with 4 patients (40%) achieving 0 staining and an absence of EM deposits. Control of C3 dysregulation was further confirmed with the observed increase in serum C3 and reduction in plasma sC5b9 levels, which is consistent with the decreased breakdown of C3. The direct inhibition of disease pathogenesis parallels improvements in histological features, as seen by reduction of inflammation and disease activity, which indicates kidney recovery and reduced disease progression.^27^^,^^28^ These results have not been previously demonstrated with proximal or terminal complement blockade. Taken together, these improvements suggest that by directly inhibiting C3 and C3b in all 3 complement pathways, pegcetacoplan may target the underlying mechanism of disease in both C3G and primary IC-MPGN and make the treatment goal of prolonged renal survival a possibility.

To date, changes in C3 staining intensity and histologic activity index have never been studied with regard to impact on C3G or primary IC-MPGN disease course, primarily because no therapy has been shown to effect such changes until recently. However, the outcomes of this trial will be useful in predicting the long-term disease-modifying effect of complement-directed therapies. Likewise, the histologic activity index has been validated as a prognostic marker for kidney outcomes in C3G but not in primary IC-MPGN.^29^ Because serial biopsies are not commonly completed outside of research settings, longitudinal changes in activity have not been formally studied until this and other ongoing clinical trials that use repeat biopsies to assess treatment effect and possible disease modification.

This study has several limitations. First, the small sample size limits generalizability of the results. Second, due to the short follow-up reported here, the long-term outcomes associated with pegcetacoplan in the posttransplant population are being further evaluated in NOBLE as secondary end points at week 52. In addition, the open-label study design offers inherent limitations, including the possibility of patient bias and the placebo effect.

In summary, for patients with C3G and primary IC-MPGN with posttransplant recurrence, 12 weeks of treatment with pegcetacoplan resulted in statistically significant reduction of C3c staining on renal biopsy and a reduction in C3G activity score. In addition, pegcetacoplan treatment was associated with a clinically important reduction in proteinuria, stable eGFR, a reduction in plasma sC5b-9, and an increase in serum C3. Pegcetacoplan was generally well-tolerated, with most TEAEs of mild-to-moderate severity. Together, these data indicate that pegcetacoplan targets the underlying pathophysiology of C3 dysregulation, reduces disease activity, and is well-tolerated in kidney transplant recipients with recurrent C3G or primary IC-MPGN. The efficacy and safety of pegcetacoplan will be further evaluated at week 52 of NOBLE and in the phase 3 VALIANT (NCT05067127) trial, which will include patients with native and posttransplant disease.

## Disclosure

ASB has received consulting fees from Amgen, Apellis, Catalyst, Genentech, Kezar, Novartis, Q32, Silence Therapeutics, and Visterra. DK has received consultancy income from Alexion, AstraZeneca, Novartis, Apellis, Gyroscope Therapeutics, Roche, Purespring Therapeutics, Samsung, Chemocentryx, Amgen, Silence Therapeutics, and Sarepta. DK is an author of patent applications referencing recombinant complement factor I production and/or formation of the C3b/FH/FI trimolecular complex. GR has received consulting fees from BioCryst and Silence Therapeutics and speakers’ bureau fees from Novartis. ZW and ZA are employees of Apellis. FF has received consulting fees from Alexion, Apellis, AstraZeneca, Biocryst, Novartis, Roche, and Sobi. MCP has received consulting fees from Alexion, Achillion, Annexon, Apellis, Biocryst, ChemoCentryx, Complement Therapeutics, Gemini, Gyroscope, MIRNA Therapeutics, Ormeros, and Q32bio Pharma; and is supported by a Wellcome Trust Senior Fellow in Clinical Science grant 212252/Z/18/Z. This research was supported by the National Institute for Health and Care Research (NIHR) Imperial Biomedical Research Centre. The views expressed are those of the authors and not necessarily those of the NIHR or the Department of Health and Social Care. ED has received consultancy income from Novartis and speakers’ bureaus fees from Sobi. All the other authors declared no competing interests.

## Supplementary Material

Supplementary File (PDF) **Supplementary Methods. Figure S1.** Representative C3c staining at baseline and week 12 (patient 1). **Figure S2.** Individual pegcetacoplan concentrations during study period. **Figure S3.** Individual serum C3 concentrations during study period. **Figure S4.** Individual plasma sC5b-9 concentrations during study period. **Table S1.** Changes in primary, secondary, and exploratory end points from baseline to week 12. **CONSORT checklist.**

## Footnotes

Supplementary File (PDF)

**Supplementary Methods.**
**Figure S1.** Representative C3c staining at baseline and week 12 (patient 1). **Figure S2.** Individual pegcetacoplan concentrations during study period. **Figure S3.** Individual serum C3 concentrations during study period. **Figure S4.** Individual plasma sC5b-9 concentrations during study period. **Table S1.** Changes in primary, secondary, and exploratory end points from baseline to week 12.
**CONSORT checklist.**
