# Supplementary material for: Efficacy and Safety of Pegcetacoplan in Kidney Transplant Recipients With Recurrent Complement 3 Glomerulopathy or Primary Immune Complex Membranoproliferative Glomerulonephritis
Source: Kidney Int Rep. 2024 Oct 10;10(1):87–98. doi: 10.1016/j.ekir.2024.09.030 (PMC11725963; doi:10.1016/j.ekir.2024.09.030)
Supplement: Supplementary File (PDF) — Supplementary Methods. Figure S1. Representative C3c staining at baseline and week 12 (patient 1). Figure S2. Individual pegcetacoplan concentrations during study period. Figure S3. Individual serum C3 concentrations during study period. Figure S4. Individual plasma sC5b-9 concentrations during study period. Table S1. Changes in primary, secondary, and exploratory end points from baseline to week 12. CONSORT checklist. [file mmc1.pdf]

SUPPLEMENTARY MATERIAL

Supplementary Methods

Patient selection

The intent-to-treat (ITT) set consisted of all patients who were randomized. This set was used for the summary of demographics, baseline characteristics, patient disposition, and analysis of all efficacy data. The safety set included all patients who received at least 1 dose of pegcetacoplan and the patients who were randomized into the SOC-only group; all safety analyses were conducted using the safety set. The pharmacokinetic (PK) set included all patients in the ITT set who received pegcetacoplan and had at least 1 evaluable post-dose PK measurement; all PK analyses were conducted using the PK set. The pharmacodynamic (PD) set included all patients in the ITT set who had at least 1 evaluable post-dose PD measurement; all PD analyses were conducted using the PD set.

Inclusion criteria

Participants were required to meet all the following criteria at screening to be included in the study:

1. At least 18 years of age
2. Clinical and pathologic evidence of recurrent complement 3 glomerulopathy (C3G) or primary immune complex membranoproliferative glomerulonephritis (IC-MPGN), as evidenced by all of the following:
    a. A diagnosis of C3G or IC-MPGN, with at least 2+ staining for C3c in the renal allograft, confirmed by a central pathologist, based on the screening renal allograft biopsy
    b. C3G or IC-MPGN must be primary and not secondary to another condition (e.g., infection, malignancy, monoclonal gammopathy, autoimmunity, chronic antibody-mediated rejection, chronic thrombotic microangiopathy, or a medication)
3. Stable (not improving) or worsening disease, in the opinion of the investigator, in the 2 months preceding the first dose of pegcetacoplan
4. Estimated glomerular filtration rate (eGFR) ≥15 mL/min/1.73 m^2, calculated by the Chronic Kidney Disease–Epidemiology Collaboration creatinine equation for adults
5. No more than 50% glomerulosclerosis or interstitial fibrosis on the screening renal biopsy
6. Stable regimen for recurrent C3G/IC-MPGN for at least 4 weeks prior to the screening renal allograft biopsy and from the time of the screening renal allograft biopsy until randomization
7. Had received required vaccinations against *Neisseria meningitidis*, *Streptococcus pneumoniae*, and *Haemophilus influenzae* (type B) or agreed to receive vaccinations if applicable vaccination records were not available. Vaccination was mandatory unless documented evidence existed that participants were nonresponders to vaccination.

8. Women of childbearing potential, defined as any women who had experienced menarche and who were not permanently sterile or postmenopausal, were required to have a negative blood pregnancy test at screening (and negative urine pregnancy at Visit 4) and agree to use protocol-defined methods of contraception from screening through 12 weeks after receiving last dose of pegcetacoplan
9. Men were required to agree to use protocol-defined methods of contraception and agree to refrain from donating semen from screening through 12 weeks after receiving last dose of pegcetacoplan
10. Willing and able to provide written informed consent
11. Able to understand and willing to comply with all scheduled procedures and other requirements of the study in the opinion of the investigator
12. Willing and able to self-administer pegcetacoplan or have an identified caregiver who can perform the administration

Exclusion criteria

Participants meeting any of the following criteria at screening or baseline were ineligible to participate in this study:
1. Absolute neutrophil count <1000 cells/mm^3 during screening (not including Day 1)
2. Previous treatment with pegcetacoplan
3. Evidence of rejection on the screening renal allograft biopsy that requires treatment
4. Diagnosis or history of human immunodeficiency virus (HIV), hepatitis B, or hepatitis C infection or positive serology at screening indicative of infection with any of these viruses
5. Weighed more than 100 kg at screening
6. Hypersensitivity to pegcetacoplan or to any of the excipients
7. History of meningococcal disease
8. Malignancy, except for the following:
    a. Cured basal or squamous cell skin cancer
    b. Curatively treated in situ disease
    c. Malignancy free and off treatment for ≥5 years
9. Significant renal disease in the renal allograft secondary to another condition (e.g., infection, malignancy, monoclonal gammopathy, rejection, or a medication) that would, in the opinion of the investigator, confound interpretation of the study results
10. Participation in any other investigational drug study or exposure to other investigational agent, device, or procedure within 30 days or 5 half-lives from the last dose of the investigational agent (whichever is longer) prior to screening period
11. Women who were pregnant or who were breastfeeding
12. Inability to cooperate or any condition that, in the opinion of the investigator, could increase the participant's risk by participating in the study or confound the outcome of the study
13. Evidence of drug or alcohol abuse or dependence, in the opinion of the investigator
14. Known or suspected hereditary fructose intolerance

Exploratory endpoints

For participants with baseline proteinuria above the ULN, the proportion of participants achieving at least a 50% reduction in proteinuria over time

For participants with baseline proteinuria above the ULN, the proportion of participants achieving complete clinical remission of proteinuria after 12 weeks of treatment, defined as normalization of proteinuria

Changes and percentage changes in proteinuria over time

Change over time in additional key biopsy features, including:

- Glomerular myeloid cell infiltration
- Glomerular macrophage infiltration (as measured by CD68 staining)
- Glomerular crescents (in participants with crescents)
- Mesangial expansion and hypercellularity
- Deposits by electron microscopy
- Activity score (based on C3G histologic index)
    - The C3G histologic index uses a semiquantitative scale of 0–3 for 7 markers of activity (mesangial hypercellularity, endocapillary proliferation, membranoproliferative morphology, leukocyte infiltration, cellular and/or microcellular crescent formation, fibrinoid necrosis, and interstitial inflammation), for a total activity score of 0–21
- Chronicity score (based on C3G histologic index)
    - The chronicity score of the C3G histologic index uses a semiquantitative scale of 0 to 3 for glomerulosclerosis, tubular atrophy, and interstitial fibrosis, and a scale of 0 to 1 for severity of arteriosclerosis, for a total score of 0–10

Table S1. Changes in in primary, secondary, and exploratory endpoints from baseline to Week 12

	C3c staining (OOM)		uPCR[a], mg/g			eGFR, mL/min/1.73 m^2				Serum creatinine, mg/dL				C3G histologic index: activity score			Electron microscopy deposits	
**Patient**	**Baseline**	**Week 12**	**Baseline**	**Week 12**	**Change from baseline, %**	**Baseline**	**Week 12**	**Change from baseline, %**	**Stabilization or improvement?**	**Baseline**	**Week 12**	**Change from baseline, %**	**Stabilization or improvement?**	**Baseline**	**Week 12**	**Change from baseline, %**	**Baseline**	**Week 12**
									Pegcetacoplan-treated patients									
1	3	0	818.3	142.5	–82.59	51	42	–17.6	Yes	1.7	2.0	17.65	Yes	1	0	–100	Present	Absent
2	2	0	1703.7	415.0	–75.64	66	72	9.1	Yes	1.4	1.3	–7.14	Yes	7	2	–71.43	N/A	Absent
3	3	3	203.7	238.7	17.18	65	40	–38.5	No	1.1	1.6	45.45	No	20	3	–85	Present	Present
4	3	0	506.7	953.7	88.22	42	34	–19	Yes	1.7	2.0	17.65	Yes	4	0	–100	Present	Absent
5	3	3	1910.3	834.3	–56.33	40	93	132.5	Yes	2.1	1.0	–52.38	Yes	11	5	–54.55	Present	Present
6	3	0	285.0	189.0	–33.68	55	52	–5.5	Yes	1.5	1.6	6.67	Yes	2	0	–100	Present	Absent
7	3	2	2963.7	1364.7	–53.95	35	32	–8.6	Yes	1.7	1.8	5.88	Yes	10	9	–10	Present	Present
8	3	2	1674.0	254.0[b]	–84.83	41	35	–14.6	Yes	1.6	1.8	12.5	Yes	14	5	–64.29	Present	Present
9	3	2	2185.0	996.0	–54.42	43	33[b]	–23.3	Yes	1.7	2.1[b]	23.53	Yes	15	4	–73.33	Present	Present
10	3	1	2984.0	4304.7	44.26	71	83	16.9	Yes	1.0	0.8	–20.0	Yes	0	1	-	Present	Present
									SOC-only patients									
11	2	3	509.7	938.0	84.04	44	40	–9.1	Yes	1.3	1.3	0	Yes	0	0	-	Absent	Present
12	3	3	1711.3	1637.0	–4.34	50	41	–18	Yes	1.6	1.9	18.75	Yes	0	3	-	Present	N/A
13	3	1	4723.3	4663.7	–1.26	66	61	–7.6	Yes	1.5	1.6	6.67	Yes	10	0	–100	Present	Present

C3G, complement 3 glomerulopathy; eGFR, estimated glomerular filtration rate; N/A, not available; OOM, orders of magnitude; SOM, standard of care.

[a] Measured by triplicate first-morning spot urine.

[b] Last available observation carried forward from Week 8.

Figure S1. Representative C3c staining at baseline and Week 12 (Patient 1)

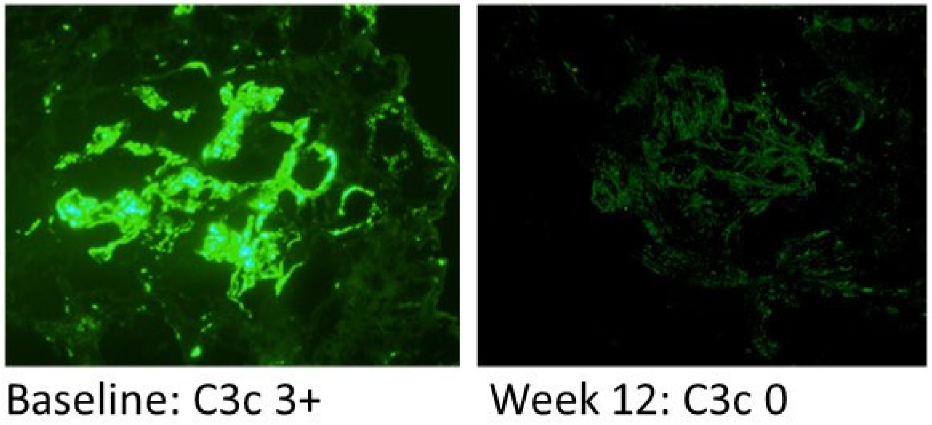

Figure S2. Individual pegcetacoplan concentrations during study period

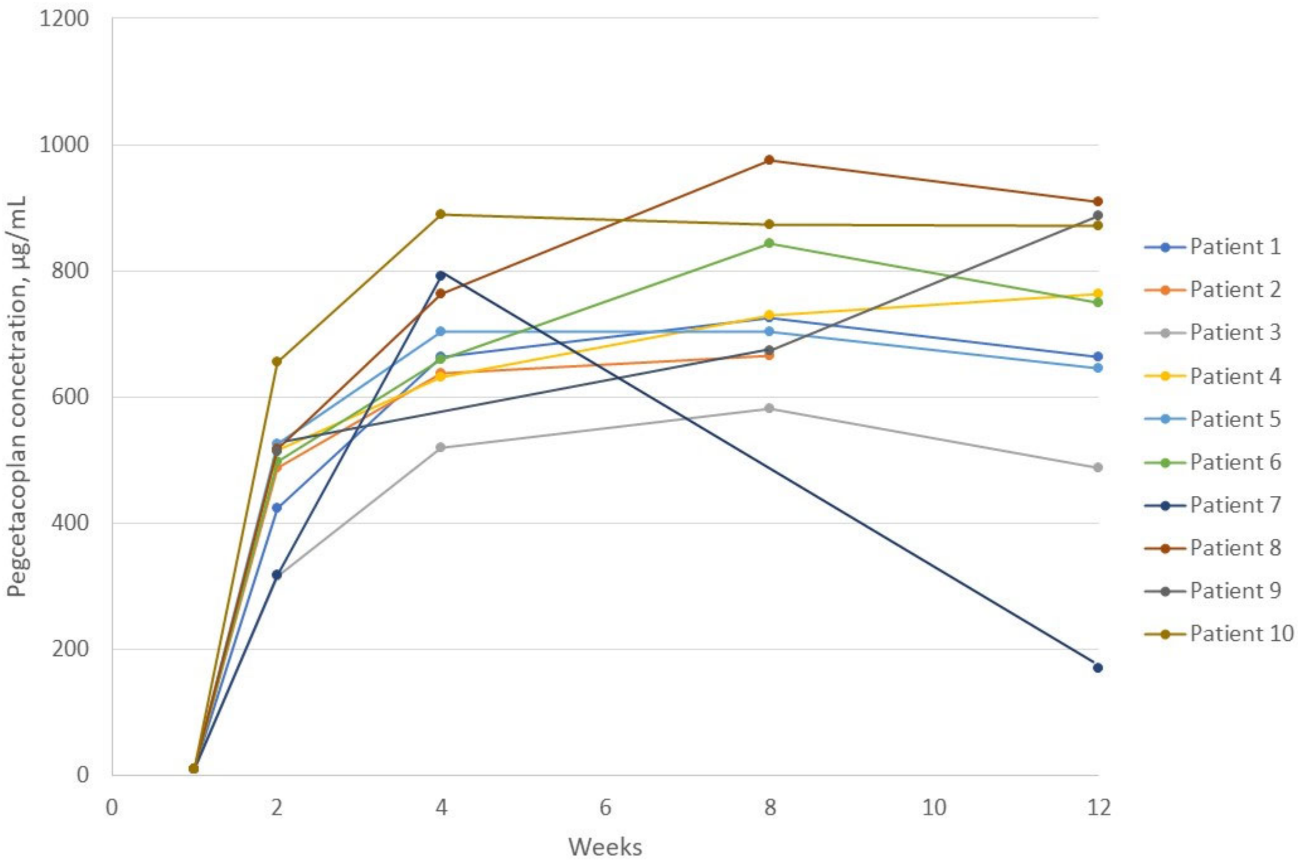

Figure S3. Individual serum C3 concentrations during study period

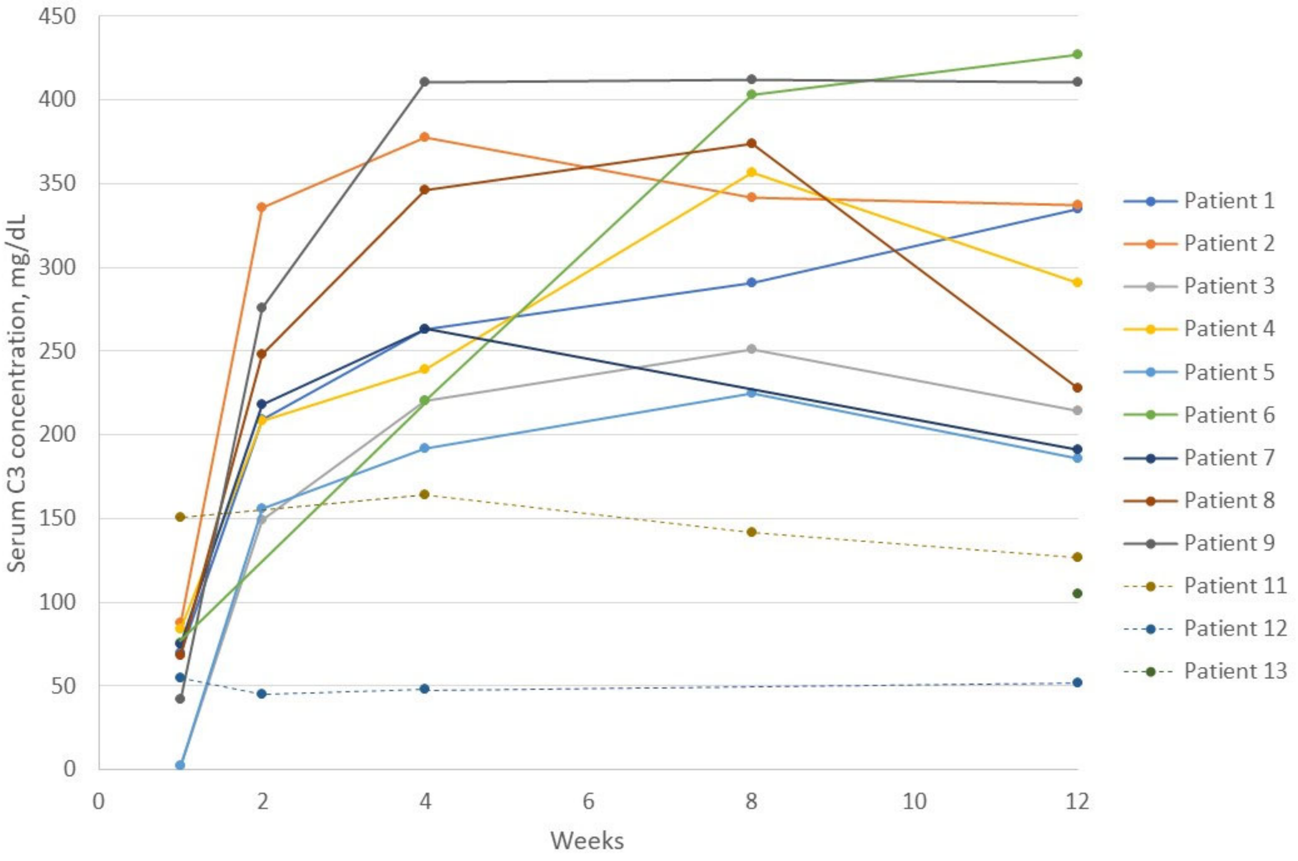

SOC, standard of care.
Note: Solid lines represent patients who received pegcetacoplan plus SOC Weeks 0–12; dashed lines represent patients who received SOC only Weeks 0–12. Patient 10 did not have available C3 concentration data. Patient 13 only had C3 data at Week 12.

Figure S4. Individual plasma sC5b-9 concentrations during study period

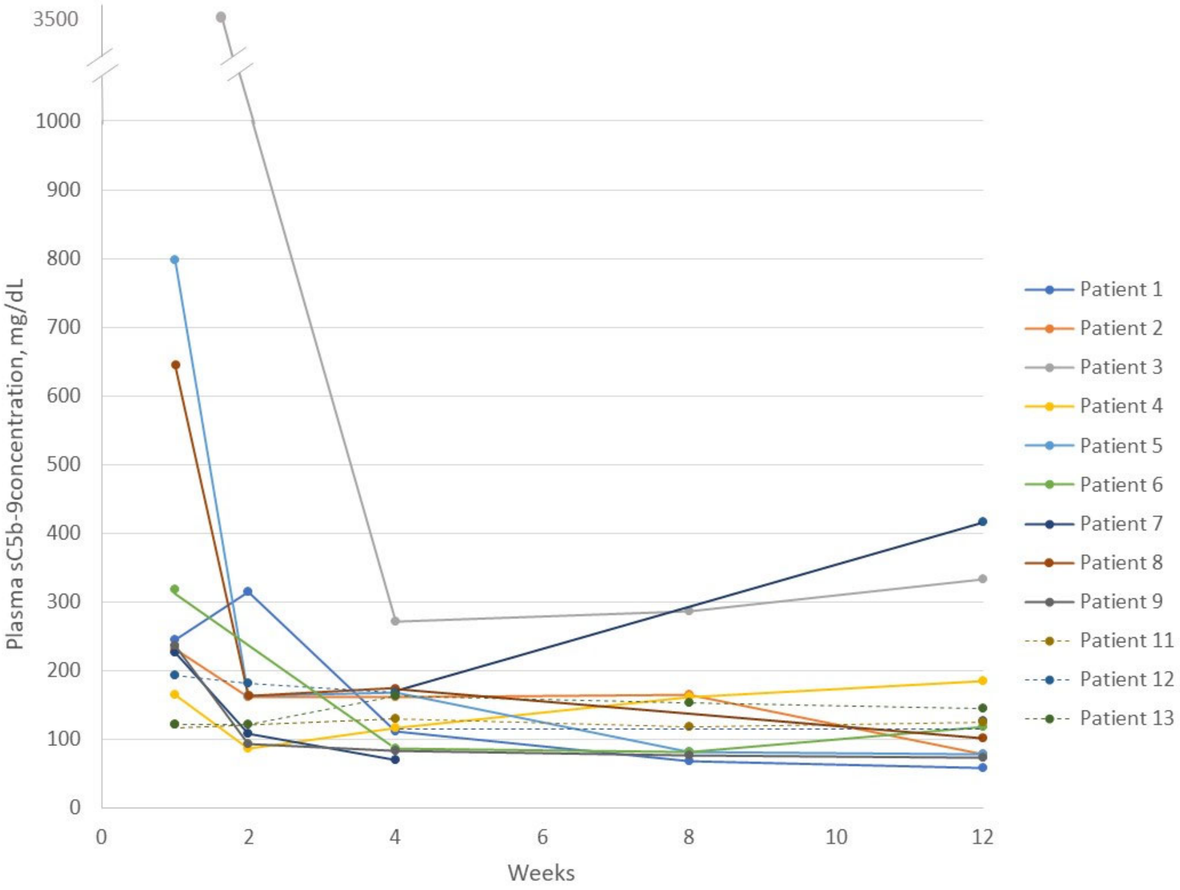

SOC, standard of care.
Note: Solid lines represent patients who received pegcetacoplan plus SOC Weeks 0–12; dashed lines represent patients who received SOC only Weeks 0–12.

CONSORT 2010 checklist of information to include when reporting a randomised trial*

Section/Topic	Item No	Checklist item	Reported on page No
**Title and abstract**			
	1a	Identification as a randomised trial in the title	1
	1b	Structured summary of trial design, methods, results, and conclusions (for specific guidance see CONSORT for abstracts)	3
**Introduction**			
Background and objectives	2a	Scientific background and explanation of rationale	5
	2b	Specific objectives or hypotheses	5
**Methods**			
Trial design	3a	Description of trial design (such as parallel, factorial) including allocation ratio	6
	3b	Important changes to methods after trial commencement (such as eligibility criteria), with reasons	N/A
Participants	4a	Eligibility criteria for participants	6, Supplement
	4b	Settings and locations where the data were collected	N/A
Interventions	5	The interventions for each group with sufficient details to allow replication, including how and when they were actually administered	6
Outcomes	6a	Completely defined pre-specified primary and secondary outcome measures, including how and when they were assessed	7, Supplement

	6b	Any changes to trial outcomes after the trial commenced, with reasons	N/A
Sample size	7a	How sample size was determined	7
	7b	When applicable, explanation of any interim analyses and stopping guidelines	N/A
Randomisation:			
Sequence generation	8a	Method used to generate the random allocation sequence	N/A
	8b	Type of randomisation; details of any restriction (such as blocking and block size)	N/A
Allocation concealment mechanism	9	Mechanism used to implement the random allocation sequence (such as sequentially numbered containers), describing any steps taken to conceal the sequence until interventions were assigned	N/A
Implementation	10	Who generated the random allocation sequence, who enrolled participants, and who assigned participants to interventions	N/A
Blinding	11a	If done, who was blinded after assignment to interventions (for example, participants, care providers, those assessing outcomes) and how	N/A
	11b	If relevant, description of the similarity of interventions	N/A
Statistical methods	12a	Statistical methods used to compare groups for primary and secondary outcomes	7
	12b	Methods for additional analyses, such as subgroup analyses and adjusted analyses	7
**Results**			
Participant flow (a diagram is strongly recommended)	13a	For each group, the numbers of participants who were randomly assigned, received intended treatment, and were analysed for the primary outcome	8, Fig 1, Fig 2

	13b	For each group, losses and exclusions after randomisation, together with reasons	8, Fig 2
Recruitment	14a	Dates defining the periods of recruitment and follow-up	N/A
	14b	Why the trial ended or was stopped	N/A
Baseline data	15	A table showing baseline demographic and clinical characteristics for each group	Table 1
Numbers analysed	16	For each group, number of participants (denominator) included in each analysis and whether the analysis was by original assigned groups	10-12
Outcomes and estimation	17a	For each primary and secondary outcome, results for each group, and the estimated effect size and its precision (such as 95% confidence interval)	10-12, Fig 1-5
	17b	For binary outcomes, presentation of both absolute and relative effect sizes is recommended	N/A
Ancillary analyses	18	Results of any other analyses performed, including subgroup analyses and adjusted analyses, distinguishing pre-specified from exploratory	N/A
Harms	19	All important harms or unintended effects in each group (for specific guidance see CONSORT for harms)	12-13, Table 2
**Discussion**			
Limitations	20	Trial limitations, addressing sources of potential bias, imprecision, and, if relevant, multiplicity of analyses	16-17
Generalisability	21	Generalisability (external validity, applicability) of the trial findings	16-17
Interpretation	22	Interpretation consistent with results, balancing benefits and harms, and considering other relevant evidence	14-16
**Other information**			

Registration	23	Registration number and name of trial registry	19
Protocol	24	Where the full trial protocol can be accessed, if available	19
Funding	25	Sources of funding and other support (such as supply of drugs), role of funders	19

Citation: Schulz KF, Altman DG, Moher D, for the CONSORT Group. CONSORT 2010 Statement: updated guidelines for reporting parallel group randomised trials. BMC Medicine. 2010;8:18.
© 2010 Schulz et al. This is an Open Access article distributed under the terms of the Creative Commons Attribution License (http://creativecommons.org/licenses/by/2.0), which permits unrestricted use, distribution, and reproduction in any medium, provided the original work is properly cited.

*We strongly recommend reading this statement in conjunction with the CONSORT 2010 Explanation and Elaboration for important clarifications on all the items. If relevant, we also recommend reading CONSORT extensions for cluster randomised trials, non-inferiority and equivalence trials, non-pharmacological treatments, herbal interventions, and pragmatic trials. Additional extensions are forthcoming: for those and for up-to-date references relevant to this checklist, see www.consort-statement.org.
